# Roadmap of DNA methylation in breast cancer identifies novel prognostic biomarkers

**DOI:** 10.1186/s12885-019-5403-0

**Published:** 2019-03-12

**Authors:** Bernardo P. de Almeida, Joana Dias Apolónio, Alexandra Binnie, Pedro Castelo-Branco

**Affiliations:** 1 Institute of Molecular Medicine, Faculty of Medicine, University of Lisbon, 1649-028 Lisbon, Portugal; 2 Department of Biomedical Sciences and Medicine, University of Algarve, Campus Gambelas, Bld. 2 - Ala Norte, 8005-139 Faro, Portugal; 3 Present address: Research Institute of Molecular Pathology (IMP), Vienna Biocenter (VBC), Vienna, Austria; 4 Centre for Biomedical Research (CBMR), University of Algarve, 8005-139 Faro, Portugal; 5 Algarve Biomedical Center, Campus Gambelas, 8005-139 Faro, Portugal; 6 William Osler Health System, Brampton, ON Canada

**Keywords:** Breast cancer, DNA methylation, Biomarkers, Diagnostic, Prognostic

## Abstract

**Background:**

Breast cancer is a highly heterogeneous disease resulting in diverse clinical behaviours and therapeutic responses. DNA methylation is a major epigenetic alteration that is commonly perturbed in cancers. The aim of this study is to characterize the relationship between DNA methylation and aberrant gene expression in breast cancer.

**Methods:**

We analysed DNA methylation and gene expression profiles from breast cancer tissue and matched normal tissue in The Cancer Genome Atlas (TCGA). Genome-wide differential methylation analysis and methylation-gene expression correlation was performed. Gene expression changes were subsequently validated in the METABRIC dataset. The Oncoscore tool was used to identify genes that had previously been associated with cancer in the literature. A subset of genes that had not previously been studied in cancer was chosen for further analysis.

**Results:**

We identified 368 CpGs that were differentially methylated between tumor and normal breast tissue (∆β > 0.4). Hypermethylated CpGs were overrepresented in tumor tissue and were found predominantly (56%) in upstream promoter regions. Conversely, hypomethylated CpG sites were found primarily in the gene body (66%). Expression analysis revealed that 209 of the differentially-methylated CpGs were located in 169 genes that were differently expressed between normal and breast tumor tissue. Methylation-expression correlations were predominantly negative (70%) for promoter CpG sites and positive (74%) for gene body CpG sites. Among these differentially-methylated and differentially-expressed genes, we identified 7 that had not previously been studied in any form of cancer. Three of these, *TDRD10*, *PRAC2* and *TMEM132C*, contained CpG sites that showed diagnostic and prognostic value in breast cancer, particularly in estrogen-receptor (ER)-positive samples. A pan-cancer analysis confirmed differential expression of these genes together with diagnostic and prognostic value of their respective CpG sites in multiple cancer types.

**Conclusion:**

We have identified 368 DNA methylation changes that characterize breast cancer tumor tissue, of which 209 are associated with genes that are differentially-expressed in the same samples. Novel DNA methylation markers were identified, of which cg12374721 (*PRAC2*), cg18081940 (*TDRD10)* and cg04475027 (*TMEM132C)* show promise as diagnostic and prognostic markers in breast cancer as well as other cancer types.

**Electronic supplementary material:**

The online version of this article (10.1186/s12885-019-5403-0) contains supplementary material, which is available to authorized users.

## Background

Breast cancer (BC) is a highly heterogeneous disease, comprising multiple histological and molecular subtypes that are associated with distinct clinical behaviours and therapeutic responses [1, 2]. Early detection and improved treatment have lead to better outcomes, however BC still ranks among the leading causes of cancer-related deaths [3]. BC has traditionally been classified based on tumor size, regional lymph node infiltration, histology, grade, and immunohistochemical evaluation of estrogen receptor (ER), progesterone receptor (PR), human epidermal growth factor receptor 2 (HER2) and proliferation marker Ki-67 [4, 5]. These factors are the most significant prognostic and therapeutic predictors in current BC clinical practice.

Recently, with the advent of high-throughput technologies, gene expression profiling has enabled a more comprehensive view of the molecular identity of breast cancer. Five major molecular and outcome related BC subtypes, known as PAM50 subtypes, were identified based on genome-wide expression analyses: Luminal-A, Luminal-B, HER-2, Normal-like and Basal-like [2, 6–8]. Breast cancer classification based on PAM50 subtypes and risk of recurrence (ROR) score have shown to significantly contribute to prognostic assessment and to facilitate more precise therapeutic decisions [9]. Other genomic tests, such as Mammaprint (Agendia, Huntington Beach, CA) and Oncotype DX (Genomic Health, Redwood City, CA) may also be used to provide prognostic and/or predictive information in early-stage breast cancer beyond the standard clinicopathological assessment and to determine the likelihood of benefit from adjuvant chemotherapy [5, 10]. Tailoring treatment to individual tumor subtypes has the potential to greatly improve breast cancer management and survival [11, 12].

Epigenetic marks, including DNA methylation, histone modifications and miRNAs, are important regulators of gene expression in normal development and disease [13, 14]. They also serve as prognostic biomarkers [15, 16] in cancer and are increasingly being investigated as therapeutic targets [17, 18]. DNA methylation involves addition of a methyl group to the cytosine pyrimidine ring in CpG dinucleotides by DNA methyltransferases (DNMTs) [19]. Canonically, promoter methylation is thought to decrease gene expression by recruitment of methyl-binding domain proteins (MBDs), that change chromatin conformation thereby preventing binding of transcription factors [15, 20, 21]. In BC, several studies have reported promoter hypermethylation leading to silencing of tumor suppressor genes, including *BRCA1* [22], E-cadherin [23] and *TMS1* [24]. However, the Wilms’ tumor suppressor 1 (*WT1*) gene is overexpressed in breast tumor tissue despite hypermethylation of its promoter [25]. Thus methylation changes in the gene promoter may correlate with either upregulation or downregulation of the associated gene [15, 20, 26, 27].

Differences in DNA methylation profiles between normal and malignant breast tissue have the potential to serve as a diagnostic and/or prognostic tool in breast cancer [21, 24, 28]. To date, most studies have examined a small number of genes [21, 22, 24], and only a few studies have performed genome-wide analyses across multiple BC subtypes [8, 29, 30]. As a result, further studies regarding genome-wide DNA methylation profiles are needed to better understand the contribution of DNA methylation patterns to breast cancer heterogeneity. Here we investigate whole genome DNA methylation patterns in BC, highlighting the potential importance of epigenetic changes in breast carcinogenesis, and identifying novel DNA methylation markers that could be useful for breast cancer classification and prognosis.

## Methods

### Datasets

Bioinformatic analyses were performed on publicly available databases including DNA methylation and gene expression data from breast tumor samples derived from The Cancer Genome Atlas Consortium (TCGA) [8] and the Molecular Taxonomy of Breast Cancer International Consortium (METABRIC) [31].

### DNA methylation and gene expression analysis

All TCGA data was retrieved from TCGA data portal (https://portal.gdc.cancer.gov/). The DNA methylation data was derived from the Illumina Infinium Human Methylation 450 k array. The methylation score for each CpG site is represented as beta values and range from 0 to 1, corresponding to unmethylated and completely methylated DNA, respectively. Gene expression data was derived from Illumina HiSeq 2000 RNA Sequencing. This dataset includes gene-level transcription estimates, expressed in RSEM normalized count.

METABRIC gene expression data was retrieved from the METABRIC dataset [31] for 1992 primary breast cancer and 144 normal tissue samples. Gene transcriptional profiling derived from the Illumina HT-12 v3 platform and data were normalized as previously described [31].

We used DAVID (http://david-d.ncifcrf.gov/) for Gene Ontology enrichment analysis.

### Gene set enrichment analyses

Genes ranked according to the coefficient of Spearman correlation were analysed for pathway enrichment using the Gene Set Enrichment Analysis software [32]. Gene sets were retrieved from the KEGG database [33, 34] and pathways with a False Discovery Rate (FDR) lower than 5% were considered significantly enriched.

### Principal component and hierarchical clustering analyses

Principal component and hierarchical clustering analyses were performed using FactoMineR [35] and gplots [36] R packages, respectively.

### OncoScore

OncoScore is a bioinformatics tool that ranks genes according to their association with cancer, based on the available scientific literature. OncoScore data was accessed on 22/06/2017 through the R package *OncoScore* [37]*,* version 1.4.2. https://github.com/danro9685/OncoScore.

### Diagnostic and prognostic value analyses

Differentially-methylated CpG sites located in the OncoScore-selected genes were analysed in terms of their diagnostic potential. The specificity and sensitivity of methylation levels for breast cancer diagnosis were evaluated by receiver-operator curve (ROC) analysis [38] with diagnostic validity suggested by an area under the ROC curve (AUC) ≥ 0.8.

To evaluate the prognostic ability of CpG sites, Kaplan-Meier survival curves were generated and log-rank *p*-value and Hazard Ratios with 95% confidence intervals were calculated [39]. Based on the AUC, a cut-off value was established for each probe in order to distinguish hypomethylated patients (blue) from hypermethylated patients (red). Optimal cut-off values were identified according to maximal sensitivity and specificity generated previously by the AUC. In addition, we performed multivariate Cox proportional-hazards model survival analyses with ER status as covariate. Only breast cancer patients with DNA methylation data and overall survival data were included in the analysis.

### Roadmap Epigenomics database analysis

Epigenomic data from normal breast myoepithelial cells was analysed using the Roadmap Epigenomics database [40] and release 9 of the Human Epigenome Atlas from the NIH Roadmap Epigenomics Mapping Consortium (http://www.roadmapepigenomics.org/data/). Data including DNA methylation levels (MeDIP), histone modification marks (ChIP), and chromatin accessibility (chromHMM) datasets. DNA methylation patterns, active histone marks H3K4me3 and H3K4me, repressive histone marks H3K27me3 and H3K9me3, and chromatin status (chromHMM) were mapped for each CpG location based on the GRCh37/hg19 genome assembly.

### Pan-cancer analysis of gene expression and CpG methylation and prognostic potential

We examined 13 cohorts from the TCGA containing both tumor and normal samples (≥ 20 samples in each group). All cohorts contained gene expression data and 12 also contained patient survival data. For each gene/CpG, we calculated the proportion of cohorts with expression results concordant with results in the breast cancer cohort, as well as methylation levels and prognostic ability in these cohorts.

### Statistical analysis

Preprocessing and normalization of data as well as all statistical analyses were performed using the R computing framework, with the exception of Kaplan-Meier survival curves, which were generated using GraphPad Prism5.0. Differential methylation and expression analyses were performed using the Mann-Whitney test, while correlation analyses were assessed using Spearman correlations. Kaplan-Meier survival curves and comparisons were performed using the log-rank test.

## Results

### Genome-wide DNA methylation analysis reveals 368 differentially methylated CpG sites in breast cancer tissue

**Fig. 1:**
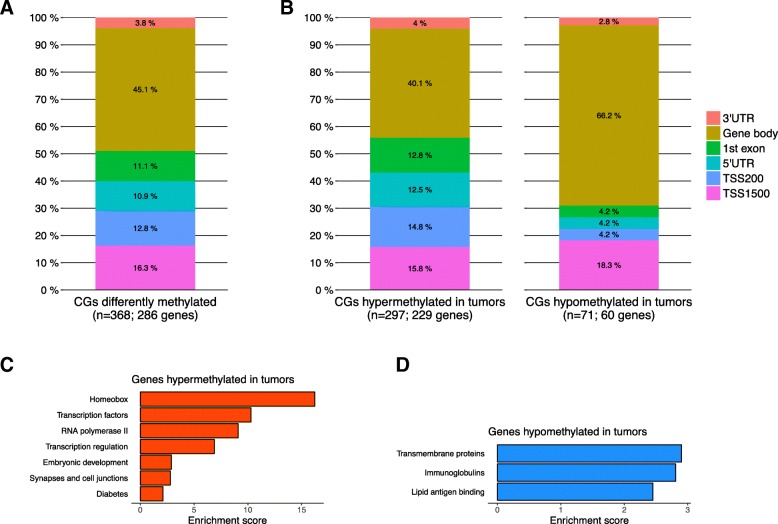
Genome-wide DNA methylation changes in breast cancer. **a** Stacked bar plot showing localization of the 368 differentially-methylated CpG sites in breast tumor tissue relative to their cognate genes. **b** Stacked bar plot showing localization of hyper- and hypomethylated CpG sites in breast tumor tissue relative to their cognate genes. The distributions are significantly different (*P* < 2.2e^− 16^, Pearson’s chi-squared test). **c** and **d** Enriched Gene Ontology categories using DAVID clustering enrichment scores for genes **c** hypermethylated or **d** hypomethylated in tumors. TSS1500, within 1500 bp of the transcriptional start site; TSS200, within 200 bp of the transcriptional start site; 5’UTR, 5′ untranslated region; 3’UTR, 3′ untranslated region

We set out to investigate the genome-wide DNA methylation profiles in a panel of 780 breast tumor samples and 83 matched normal samples from The Cancer Genome Atlas (TCGA). Although methylation of distal regions, such as enhancers, is relevant for gene regulation in breast cancer [41], we intentionally focused on proximal gene regions by limiting our analysis to CpG probes mapping to a known gene (*n* = 251,574) to facilitate the link with the respective target gene. To identify CpG sites showing the most significant and relevant tumor-specific changes in methylation, CpG’s with a ∆β (between tumors and normal tissues) equal to or greater than 0.4 were selected. We identified 368 differentially-methylated CpG sites that distinguished tumor and normal breast tissues (∆β ≥ 0.4 and FDR ≤ 5%), mapping to 286 unique genes (Fig. 1a; Additional file 1: Table S1). Hypermethylated CpG sites (80.7%) predominated in tumor tissue relative to hypomethylated sites (19.3%) (*P* < 2.2 × 10^− 16^; Fig. 1b). Hypermethylated and hypomethylated probes also localized to different areas within their associated genes (*P* = 0.001). More than 50% of hypermethylated CpG sites were localized in upstream regulatory regions including the promoter, 5′ untranslated region, and 1st exon (TSS1500, TSS200, 5’UTR and 1st exon), while only 30% of hypomethylated CpG sites localized to these regions (Fig. 1b). Conversely, hypomethylated CpG sites were localized predominantly in the gene body (66.2%), a phenomenon that has been postulated in other cancers to contribute to activation of aberrant intragenic promoters that are normally silenced [42, 43].

Functional enrichment analysis revealed that genes associated with hypermethylated CpG sites are enriched for homeobox genes and transcription factors, while those associated with hypomethylated CpG sites are enriched for transmembrane proteins and immunoglobulins (Fig. 1c-d, Additional file 2: Table S2).

### Correlation of DNA methylation with gene expression change in BC

**Fig. 2:**
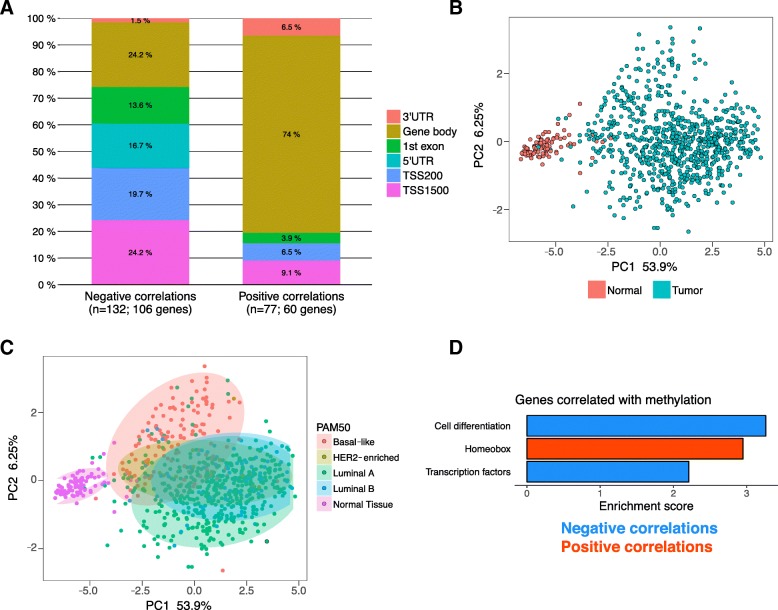
209 CpG probes are correlated with cognate gene expression. **a** Stacked bar plot showing localization of differentially-methylated CpG sites within their cognate genes subdivided by the correlation between methylation change and expression change. Negatively-correlated CpG sites are shown in the first bar, and positively-correlated CpG sites in the second bar. The distributions are significantly different (*P* < 2.2 × 10^− 16^, Pearson’s chi-squared test). **b** and **c** Principal Component Analyses using the 209 differentially-methylated probes located in differentially-expressed genes, colored by (B) sample type or (C) PAM50 subtype. **d** Enriched Gene Ontology categories using DAVID clustering enrichment scores for genes with negative (blue) or positive (red) correlations

To explore the relationship between DNA methylation and gene expression in BC, we compared the direction of CpG methylation change (hyper- vs hypomethylated) with the direction of expression change in the corresponding genes. Among the 368 differentially-methylated CpG sites, we identified 209 that were associated with differentially-expressed genes (FDR < 5%), representing a total of 164 genes. We then correlated the direction of methylation change with the direction of expression change of the cognate gene. Negative correlations (59%) predominated relative to positive correlations (41%) (*p* < 2.2 × 10^− 16^, Additional file 3: Figure S1), driven by a large number of hypermethylated CpG sites that were associated with downregulated genes (Additional file 4: Table S3). When negative and positive correlations were subdivided according to CpG location within the associated gene, > 70% of negative correlations involved CpG sites located in the upstream regulatory regions (promoter, 5’UTR, 1st exon), while 74% of positive correlations involved CpG sites found in the gene body (Fig. 2a). Thus promoter hypermethylation correlated with gene downregulation, while gene body hypermethylation correlated with gene upregulation, as previously observed in a separate genome-wide study [29].

We next analyzed the same 209 CpG sites (associated with differentially-expressed genes) to ascertain the sources of variability at these methylation sites. Principal Component Analysis confirmed that sample type (normal breast vs breast tumor) is the primary source of variability underlying the methylation signature, accounting for 53.9% of variability (Fig. 2b). The second component (6.25%) was putatively explained by the PAM50 subtypes within the breast tumors as identified in TCGA (Fig. 2c), with higher Principal Component 2 values associated with basal breast tumors and poorer outcomes (*P* = 0.01, Log-rank test, Additional file 3: Figure S2). Unsupervised hierarchical clustering, using the same 209 CpG probes, revealed the existence of two major groups, however, these did not show obvious clustering of clinical traits (Additional file 3: Figure S3).

Functional enrichment analysis of the 164 differentially-methylated and differentially-expressed genes revealed enrichment for homeobox genes (positively correlated with methylation change, upregulated expression) as well as transcription factors (negatively correlated with methylation change, downregulated expression) and cell differentiation genes (negatively correlated with methylation change) (Fig. 2d, Additional file 2: Table S2).

### METABRIC validation and OncoScore analysis reveal 7 new genes related to BC

To validate our gene expression results we used transcriptomic data from the METABRIC dataset [31], which comprises 1992 breast tumor samples and 144 normal adjacent tissues. We were able to validate 88 of the 164 genes (53.7%) as differently expressed in breast tumor tissue relative to normal tissue, with the direction of expression change being concordant between the datasets (Additional file 5: Table S4). Of the remaining 76 genes, 68 genes did not show differential expression in the METABRIC dataset while no data was available for the final 8 genes.

We next determined which of the 96 differentially-methylated genes with validated (88) or unconfirmed (8) gene expression changes had previously been associated with cancer in the medical literature. We used the OncoScore tool [37], a text-mining algorithm that ranks genes according to their appearance in the cancer literature, to analyse the 96 genes. The top ranked gene, *WT1*, had an Oncoscore of 77.5 while 81 genes had Oncoscores ≥1, indicating at least one citation in a cancer-related article (Additional file 6: Table S5). A total of 7 genes had Oncoscores of 0, indicating no prior association with cancer in the medical literature. No Oncoscore data was available for 8 genes.

**Fig. 3:**
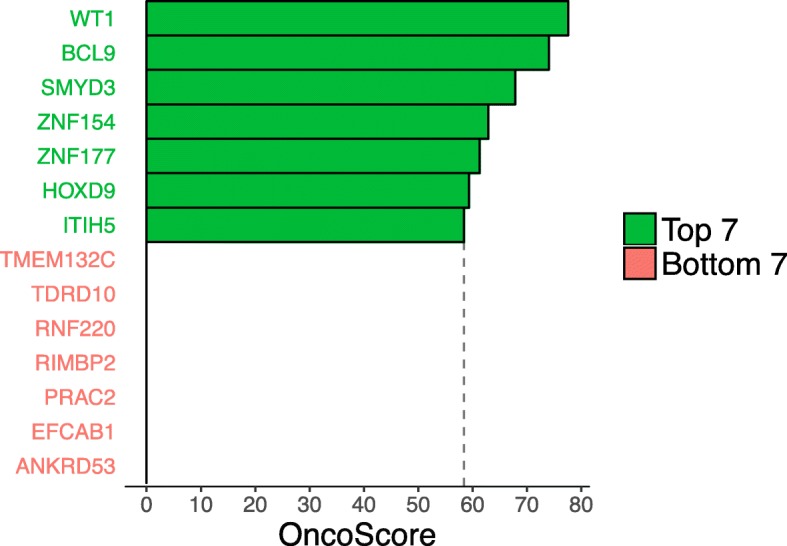
OncoScore of the “top 7” (green) and “bottom 7” (red) genes

After Oncoscore analysis we selected the top 7 genes (strongly associated with cancer: *WT1*, *BCL9*, *SMYD3*, *ZNF154*, *ZNF177*, *HOXD9*, and *ITIH5*) and the bottom 7 genes (no published association with cancer: *TMEM132C*, *TDRD10, RNF220, RIMBP2, PRAC2* (*C17orf93*)*, EFCAB1*, and *ANKRD53*) for further analysis of diagnostic and prognostic potential (Fig. 3).

### Identification of candidate diagnostic and prognostic biomarkers in breast cancer

Within the 14 genes selected for closer analysis, 18 differentially-methylated CpGs were identified (Table 1). These CpG sites were analysed for diagnostic and prognostic potential using the area under the ROC curve (AUC) method [38] and Kaplan-Meir survival curves, respectively.

**Table 1:** List of the Top and Bottom 7-ranking methylation markers selected as potential biomarkers

	CpG ID	Gene	∆β methylation (tumor - normal)	Correlation (methylation-expression)	AUC	Overall Survival
Top 7	cg10244666	*WT1*	0.44; *p* = 2.57e-40	r:0.17; *p* = 1.09e-6	0,9430 (CI:0.9279–0.9582); *p* < 0.0001	ns
	cg03441279	*BCL9*	−0.41; *p* = 5.8e-25	r:-0.32; *p* = 1.58e-19	0,8434 (CI:0.8184–0.8684); *p* < 0.0001	ns
	cg25025181	*SMYD3*	−0.45; *p* = 6.18e-39	r:-0.31; *p* = 2.36e-19	0,9324 (CI:0.9163–0.9486); *p* < 0.0001	ns
	cg01268824	*ZNF154*	0.42; *p* = 4.68e-34	r:-0.63; *p* = 1.44e-85	0,9002 (CI:0.8778–0.9226); *p* < 0.0001	*p* = 0.0097
	cg09578475	*ZNF177*	0.51; *p* = 4.87e-40	r:0.20; *p* = 2.73e-8	0,9378 (CI:0.9219–0.9537); *p* < 0.0001	ns
	cg08065231		0.46; *p* = 9.51e-39	r:0.23; *p* = 1.54e-10	0,9320 (CI:0.9153–0.9486); *p* < 0.0001	ns
	cg13703871		0.47; *p* = 2.91e-40	r:0.17; *p* = 3.13e-6	0,9410 (CI:0.9257–0.9562); *p* < 0.0001	ns
	cg22674699	*HOXD9*	0.40; *p* = 3.15e-28	r:-0.17; *p* = 1.12e-6	0,8679 (CI:0.8427–0.8931); *p* < 0.0001	*p* = 0.0381
	cg10119075	*ITIH5*	0.41; *p* = 1.51e-39	r:-0.26; *p* = 1.73e-13	0,9397 (CI:0.9243–0.9552); *p* < 0.0001	ns
Bottom 7	cg15165122	*ANKRD53*	0.41; *p* = 2.09e-34	r: −0.48; *p* = 2.34e-46	0,9028 (CI:0.8827–0.9229); *p* < 0.0001	ns
	cg12743248	*EFCAB1*	−0.45; *p* = 5.39e-45	r: 0.44; *p* = 8.49e-38	0,9664 (CI:0.9553–0.9775); *p* < 0.0001	ns
	cg12374721	*PRAC2*	0.46; *p* = 9.42e-36	r:0.39; *p* = 1.63e-30	0,9118 (CI:0.8923–0.9313); *p* < 0.0001	*p* = 0.0134
	cg27170427	*RIMBP2*	−0.46; *p* = 1.24e-46	r:0.35; *p* = 1.79e-24	0,9766 (CI:0.9680–0.9851); *p* < 0.0001	ns
	cg17192862		−0.41; *p* = 1.29e-46	r:0.45; *p* = 6.74e-40	0,9765 (CI:0.9675–0.9856); *p* < 0.0001	ns
	cg10224098	*RNF220*	0.45; *p* = 1.01e-39	r:-0.09; *p* = 1.51e-2	0,9393 (CI:0.9220–0.9566); *p* < 0.0001	ns
	cg18081940	*TDRD10*	0.41; *p* = 1.58e-39	r:-0.20; *p* = 4.17e-8	0,9360 (CI:0.9189–0.9531); *p* < 0.0001	*p* = 0.0037
	cg10216717	*TMEM132C*	−0.45; *p* = 2.02e-49	r:0.46; *p* = 3.44e-42	0,9920 (CI:0.9872–0.9968); *p* < 0.0001	ns
	cg04475027		0.42; *p* = 1.19e-40	r:-0.23; *p* = 3.24e-11	0,9446 (CI:0.9289–0.9604); *p* < 0.0001	*p* = 0.0291

*ns* not significant

The “Top 7” and “Bottom 7” genes (based on OncoScore results) selected for analysis as potential methylation biomarkers

Within the “top 7” genes, there were 9 differentially-methylated CpG sites, of which 7 were hypermethylated and 2 hypomethylated (Table 1). All 9 CpG sites were able to distinguish breast tumor tissue from normal tissue (AUC > 0.8 and *p* < 0.0001; Table 1). Only 2 CpG sites showed an association with poor prognosis. These were both hypermethylated CpG sites located in the promoters of the *ZNF154* and *HOXD9* genes respectively that were negatively correlated with gene expression (*ZNF154: p* = 0.0097 and *HOXD9: p* = 0.0266, Additional file 3: Figure S4). When the different ER status were taken into account as covariates in a multivariate analysis, only the HOXD9 CpG methylation remained significantly associated with poor prognosis (*p* = 0.02, Additional file 3: Figure S4E,F). These findings suggest that silencing of these genes by DNA methylation may have negative implications for prognosis, which is in accordance with previous data from triple negative breast cancer [44] and metastatic melanoma [45].

**Fig. 4:**
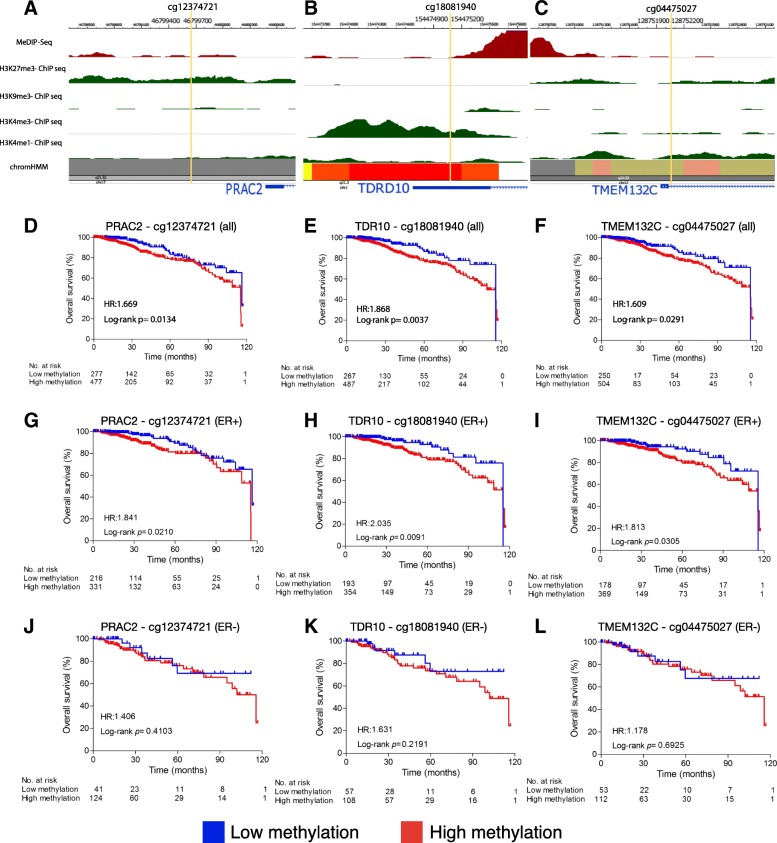
Epigenetic analysis of CpGs sites from *PRAC2*, *TDRD10* and *TMEM132C* (“bottom 7” genes). **a** MeDIP-Seq data shows that cg12374721 (*PRAC2*) is hypomethylated in normal breast cells. ChIP-Seq data shows enrichment of H3K27me3 histone repressive marks (green peaks) and lack of H3K4me1 and H3K4me3 active histone marks. ChromHMM classified this region as a poly comb repressive region (grey color). **b** cg18081940 (*TDRD10*) and **c** cg04475027 (*TMEM132C*) sites are hypomethylated in normal cells and overlap with open chromatin and H3K4me1 and H3K4me3 histone modification peaks associated with active transcription (green peaks). ChromHMM classified **b** cg18081940 (*TDRD10*) region as an active TSS (red) and **c** cg04475027 (*TMEM132C*) as a bivalent enhancer (dark yellow). **d-l** Kaplan-Meier curves for the CpG probes located in **d** *PRAC2*, **e** *TDRD10* and **f** *TMEM132C* showed that hypomethylation is associated with better overall survival. Hypomethylation of the 3 CpGs was also associated with better prognosis in ER-positive samples (**g-i**), but not in ER-negative samples (**j-l**). Based on the AUC, a cut-off value was established for each probe in order to distinguish hypomethylated patients (blue) from hypermethylated patients (red). The following cut-offs were used: **d** 0.5503, corresponding to the 37th percentile (PRAC2-cg12374721); **e** 0.5243, 35th percentile (TDRD10-cg18081940); **f** 0.4014, 33rd percentile (TMEM132C-cg04475027); **g** 0.5503, 39th percentile; **h** 0.5243, 35th percentile; **i** 0.4014, 33rd percentile; **j** 0.5503, 25th percentile; **k** 0.5243, 35th percentile; **l** 0.4014, 32th percentile

Within the “bottom 7” genes not previously associated with cancer there were a further nine differentially-methylated CpG sites (5 hypermethylated, 4 hypomethylated) (Table 1). All 9 CpG sites were able to distinguish breast tumor tissue from normal tissue (AUC > 0.8 and *p* < 0.0001, Table 1). Site cg10216717, located in gene *TMEM132C*, showed the highest discriminative accuracy with an AUC of 0.9920 (Table 1). Only 3 CpG sites showed an association with poor prognosis (Fig. 4). Site cg12374721 (*PRAC2* gene) was hypermethylated in breast tumor tissue and positively correlated with gene expression (*p* = 0.0134, Fig. 4d). Sites cg18081940 (*TDRD10* gene) and cg04475027 (*TMEM132C* gene) were also hypermethylated but were negatively correlated with gene expression (*p* = 0.0037 and *p* = 0.0291 respectively, Fig. 4e, f). All 3 CpG sites were associated with poor prognosis in ER-positive breast cancer samples, but none in ER-negative (Fig. 4g-l). The overall association of TDRD10 and TMEM132C’s CpG sites remained significant when ER status was taken into account as covariate in a multivariate analysis (*p* = 0.06 and 0.03, respectively, Additional file 3: Figure S5). When a combined signature of these 3 CpG sites was analysed, patients with a higher hypermethylation index showed poorer overall prognosis (*p* = 0.02; HR: 1.853; Additional file 3: Figure S6). These data suggest a possible role for *PRAC2* (increased expression in tumor tissue) as an oncogene and *TDRD10* and *TMEM132C (*decreased expression in tumor tissue) as tumor suppressor genes.

### Roadmap of epigenomic regulatory elements

We used the Roadmap Epigenomics database [40] to analyze the 5 CpG sites that showed both diagnostic and prognostic potential in BC. Using data from normal breast myoepithelial cells, we plotted DNA methylation status, histone modification marks and chromatin accessibility (chromHMM) data for these CpG sites and their associated genes.

Sites cg01268824 (*ZNF154*), cg22674699 (*HOXD9*), cg18081940 (*TDRD10*), and cg04475027 (*TMEM132C*) localized to gene promoter regions, were hypermethylated, and were negatively correlated with expression in breast tumor tissue, suggesting that DNA methylation at these sites may silence gene transcription (Table 1). At all 4 of these CpG sites Roadmap Analysis revealed that in normal breast cells low methylation levels was associated with open chromatin and active histone modification marks, namely H3K4me1 and H3K4me3 (Fig. 4b and c, Additional file 3: Figure S4). Accordingly, hypermethylation of these CpG sites may hinder the binding of transcription factors or enhancers and/or modify chromatin accessibility leading to gene silencing in breast cancer.

Conversely, site cg12374721 (*PRAC2*) was hypermethylated and positively correlated with gene transcription in tumor tissue (Table 1). Roadmap analysis revealed that cg12374721 was located in a polycomb repressive region in normal breast myoepithelial cells, which is associated with repressive chromatin marks, including enrichment of H3K27me3 marks (facultative heterochromatin) and lack of H3K4me1 and H3K4me3 (Fig. 4a). Therefore, the gain of methylation in this CpG may contribute to transcriptional activation by inhibiting the binding of transcriptional repressors or altering the repressive chromatin conformation in cancer.

### Identification of 3 new breast cancer-related genes

Genes *PRAC2*, *TDR10* and *TMEM132C* showed differential methylation and differential expression in breast tumor samples relative to normal breast tissue and also contained CpG sites showing diagnostic and prognostic value in breast cancer. None of these genes has previously been reported in the cancer literature. *PRAC2* is upregulated in breast tumor tissue whereas *TDR10* and *TMEM132C* are both downregulated.

**Fig. 5:**
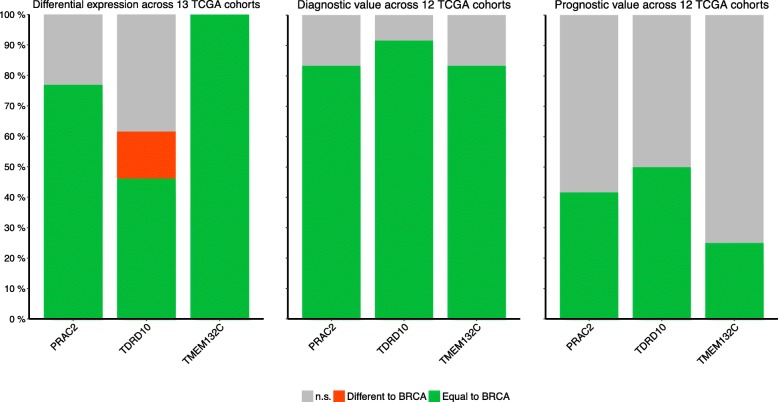
Pan-cancer analysis of CpGs sites from *PRAC2*, *TDRD10* and *TMEM132C*. Bar plots showing the proportion of cohorts with results concordant with breast cancer (green), opposite to breast cancer (red) or non-significant (grey)

We further analyzed expression of these 3 genes in 13 non-breast cancer TCGA cohorts including colorectal adenocarcinoma, head and neck cancer, hepatocellular carcinoma, lung adenocarcinoma, lung squamous cell carcinoma, prostate adenocarcinoma, and thyroid carcinoma (Additional files 7 and 8: Table S6 and S7). Expression of *TMEM132C* was downregulated across all 13 non-breast cancer cohorts while *PRAC2* was upregulated in 77% of cohorts. *TDRD10* was downregulated in 46% of cohorts (similar to BC) but was upregulated in kidney clear cell carcinoma and thyroid carcinoma cohorts (Fig. 5). We further analysed the diagnostic ability of the 3 CpG sites associated with these genes in non-breast cancer cohorts. All 3 sites correlated with cancer diagnosis in 10 or more of the 12 TCGA cohorts containing methylation data (Fig. 5). Correlation with survival was identified in 50% (*TDRD10*), 42% (*PRAC2*), and 25% (*TMEM132C*) of the 12 TCGA cohorts, with no significant opposing results (Fig. 5). None of the 3 CpGs sites showed diagnostic or prognostic potential in the thyroid carcinoma cohort, suggesting that these pathways are not important for the pathogenesis of this particular cancer.

## Discussion

DNA methylation is an important epigenetic alteration that can modify gene expression and is commonly perturbed in cancer [14]. Its impact on aberrant gene expression in breast cancer remains poorly understood. Here we report a roadmap of DNA methylation changes in breast cancer and their association with gene expression changes in matched samples. Using a breast cancer cohort from TCGA we identified 368 individual CpG sites that were differentially methylated between tumor and normal breast tissue. A majority of sites were hypermethylated and located in upstream transcriptional regulatory regions, including the promoter. This finding is in agreement with previous studies reporting promoter hypermethylation as a mechanism of tumor suppressor gene silencing in breast cancer [46]. Functional analysis revealed that the hypermethylated gene set was enriched for homeobox genes and transcription factors. Homeobox genes have previously been reported as differently methylated in breast cancer [47], as well as in other cancer types [48]. Hypomethylated CpG sites were located primarily in the gene body, consistent with intragenic DNA hypomethylation as a feature of many tumors, where it enables spurious transcription initiation and consequent abnormal transcripts [42, 43].

Our results also confirm that DNA methylation is strongly associated with repression of gene expression in breast cancer. A majority of the 209 CpG sites located in differentially-expressed genes showed negative correlations between the direction of methylation change and the direction of expression change. These CpG sites were located primarily in upstream transcriptional regulatory regions. Conversely, CpG sites showing positive correlations with direction of gene expression change were found primarily in the gene body. Functional enrichment of these latter genes was positive for homeobox genes. Further studies are required to elucidate the role of DNA methylation in the regulation of this important class of genes.

Using the METABRIC [31] dataset we were able to validate the direction of expression change in 88 of the differentially-methylated genes. The OncoScore tool [37] was used to identify which of these genes (along with 8 genes that did not appear in the METABRIC data) had previously been associated with cancer in the medical literature. We then selected the 7 genes with the highest OncoScores and 7 genes with the lowest OncoScores to analyze their associated CpG sites as potential diagnostic and prognostic biomarkers in breast cancer. Intriguingly, all of the CpG sites in all 14 genes, including those not previously associated with cancer, were able to accurately distinguish breast tumor and normal tissue (AUC > 0.8 and *p* < 0.0001, Table 1). The highest discriminative accuracy was shown by site cg10216717 located in the *TMEM132C* gene (Table 1). Furthermore, 3 CpGs located in genes not previously associated with cancer, *PRAC2*, *TDRD10* and *TMEM132C*, were able to predict breast cancer overall survival, and more particularly survival of ER-positive patients (Table 1, Fig. 4), suggesting their potential as diagnostic and prognostic markers in BC.

The *PRAC2* gene is located between the *HOXB13* and *PRAC* genes, both of which encode small nuclear proteins. *PRAC2* is highly expressed in prostate tissue and has been suggested to play a role in prostate growth and development [49]. For this reason PRAC2 was given the name “Prostate Cancer Susceptibility Candidate 2” gene. However, it has not previously studied or associated with any type of cancer [37]. In the TCGA dataset, *PRAC2* was highly expressed in breast tumor tissue relative to normal tissue (Additional file 5: Table S4). Methylation of its associated CpG site, cg12374721, which is located in the gene promoter, was positively correlated with gene transcription in tumor tissue. This contradicts one of the central paradigms of DNA methylation, namely that promoter methylation results in gene silencing [20]. Analysis of data from the Roadmap Epigenetics Atlas shows enrichment of H3K27me3 in this region in normal breast cells, a histone mark that is associated with repressive chromatin. Thus methylation of this site in breast tumor tissue may contribute to *PRAC2* transcriptional activation by blocking the binding of transcriptional repressors. Additionally, hypermethylation of site cg12374721 was associated with reduced survival (Table 1, Fig. 4d). This may suggest an oncogenic role for *PRAC2* in BC, as has been suggested in prostate cancer [49].

Unlike *PRAC2*, genes *TDRD10* and *TMEM132C* are both downregulated in breast tumor tissue when compared to normal tissue (Additional file 5: Table S4). Their hypermethylated CpG sites, cg18081940 (*TDRD10* 5’UTR) and cg04475027 (*TMEM132C* gene body), are negatively correlated with gene expression (Table 1). Methylation of both of these sites is also associated with reduced survival (Fig. 4e and f), independent of ER status (Additional file 3: Figure S5). Analysis of histone marks in normal breast tissue reveals that cg18081940 (*TDRD10*) and cg04475027 (*TMEM132C*) both overlap with open chromatin and histone modification marks associated with enhancers (H3K4me1 and H3K4me3) (Fig. 4b and c). Accordingly, hypermethylation of these CpGs may hinder the binding of transcription activators leading to gene silencing in breast cancer, suggesting a tumor suppressor function for those genes. *TDRD10* (Tudor domain containing 10) is a member of the TDRD protein family, that binds to methylated arginine/lysine residues and plays a crucial role in chromatin and transcriptional regulation, genome stability and RNA metabolism [50, 51]. Dysregulation of TDRDs has been reported in BC. Surprisingly, a negative correlation has been observed between DNA copy number and mRNA expression for *TDRD10*, demonstrating its importance in suppressing carcinogenesis [50]. Finally, the *TMEM132C* (Transmembrane Protein 132C) gene belongs to a family of five TMEM132 proteins, which are associated with hearing loss, panic disorder and cancer [52, 53]. However, the biological function of these genes is still under investigation and as yet there is no scientific literature relating to *TMEM132C*.

In addition to the identification of *PRAC2*, *TDR10* and *TMEM132C* as novel DNA methylation-gene markers in breast cancer, analysis of their expression and diagnostic and prognostic potential revealed they may also be relevant in other cancer types (Fig. 5). Interestingly, in thyroid carcinoma, which is a relatively indolent tumor, none of the 3 CpGs analyzed showed diagnostic or prognostic potential (Additional file 7: Table S7). Thus *PRAC2*, *TDR10* and *TMEM132C* may be more relevant in rapidly growing cancers. These genes merit further study to better understand their role in breast cancer pathogenesis. Moreover, validation of these and other DNA methylation-based diagnostic and prognostic markers may have significant clinical benefits, namely in terms of sample stability and cost when compared to RNA-based tests (eg. Oncotype and Mammaprint) [5, 10].

## Conclusion

We have investigated DNA methylation patterns in BC using a genome-wide approach and have correlated methylation changes with gene expression data from TCGA and METABRIC datasets. This work provides a landscape of aberrant DNA methylation changes in breast cancer and their association with gene expression regulation. Both positive and negative correlations were observed, suggesting that both CpG hypermethylation and hypomethylation may be crucial events in breast carcinogenesis. Three novel DNA methylation-gene candidate biomarkers for breast cancer were identified and validated in other cancer datasets. Sites cg12374721 (*PRAC2*), cg18081940 (*TDRD10*) and cg04475027 (*TMEM132C*) may be effective as diagnostic and prognostic tools not only in breast cancer but also in other cancer types.

## Additional files

Additional file 1: **Table S1.** List of CpGs differently methylated between breast cancer and matched-normal samples. (XLSX 61 kb)

Additional file 2: **Table S2.** Results of DAVID clustering Gene Ontology analyses. (XLSX 95 kb)

Additional file 3: **Figure S1.** Genome-wide impact of DNA methylation on gene expression. Distribution of Spearman correlation coefficients (SCC) between DNA methylation and cognate gene expression levels (59% of negative SCC; *P* < 2.2 × 10^− 16^, 1-sample proportions test). **Figure S2.** Higher values of principal component 2 are associated with poorer survival. Kaplan Meier (KM) curve showing patients subdivided by principal component 2 value with a cutoff of 0.095 (*p* = 0.01, Log-rank test). **Figure S3.** Heatmap showing hierarchical clustering analysis of 209 differentially-methylated CpG sites associated with 164 differentially-expressed genes. **Figure S4.** 2CpGs from ZNF154 and HOXD9 are epigenetically dynamic and predict prognostic. Upper panel- (A) cg01268824-ZNF154 and (B) cg22674699-HOXD9 sites are hypomethylated in normal cells, overlapping with open chromatin and active histone modification marks (H3K4me1 and H3K4me3, green peaks). ChromHMM classified (A) cg01268824 region as an active TSS (red) and (B) cg22674699 as a bivalent enhancer (dark yellow). Middle panel- KM curves evidenced that hypomethylation of CpGs located in (C) ZNF154 and (D) HOXD9 are associated with a longer overall survival. Cut-offs of 0.6188, (50th percentile of ZNF154-cg01268824) and 0.6102 (49th percentile of HOXD9-cg22674699) were used. Bottom panel- Forest plot of Cox multivariate survival analyses of methylation values of (E) ZNF154 and (F) HOXD9 CpG probes with ER status as covariate. **Figure S5.** Cox multivariate analyses of the 3 CpGs sites from “bottom 7” genes in BC. Forest plot of Cox multivariate survival analyses of methylation values of (A) PRCA2, (B) TDRD10 and (C) TMEM132C CpG probes with ER status as covariate. **Figure S6.** Prognostic signature of the 3 CpGs sites from “bottom 7” genes in BC. KM curve for the combined signature of the 3 CpGs sites correspondent to PRAC2-cg12374721, TDRD10-cg18081940 and TMEM132Ccg04475027. Low methylation levels was significantly associated with better prognosis (*p* = 0.02; HR: 1.853). (PDF 2734 kb)

Additional file 4: **Table S3.** List of CpGs located in genes that are differentially expressed between breast cancer and matched-normal TCGA samples and whose methylation levels are correlated with cognate gene expression. (XLSX 61 kb)

Additional file 5: **Table S4.** List of genes differently expressed between normal and tumor METABRIC samples and with concordant results with TCGA breast cancer analyses. (XLSX 39 kb)

Additional file 6: **Table S5.** Results of OncoScore analyses for the 164 differentially-methylated and differentially-expressed genes. (XLSX 35 kb)

Additional file 7: **Table S6.** Results of pan-cancer analyses of differential gene expression of *PRAC2*, *TDRD10* and *TMEM132C*. (XLSX 35 kb)

Additional file 8: **Table S7.** Results of pan-cancer analyses of diagnostic and prognostic potential of the CpGs from *PRAC2*, *TDRD10* and *TMEM132C*. (XLSX 31 kb)

## Abbreviations

450 K: Illumina Infinium HumanMethylation 450 K BeadChip Array
AUC: Area under the ROC curve
ChIP: Chromatin immunoprecipitation
chromHMM: Chromatin Hidden Markov Model
CpG: Cytosine-phosphate-guanine
FDR: False discovery rate
HR: Hazards ratio
MeDIP: Methylated DNA immunoprecipitation
NIH: National Institutes of Health
PAM50: Prediction Analysis of Microarray 50
TCGA: The Cancer Genome Atlas
