# Supplementary material for: Roadmap of DNA methylation in breast cancer identifies novel prognostic biomarkers
Source: BMC Cancer. 2019 Mar 12;19:219. doi: 10.1186/s12885-019-5403-0 (PMC6416975; doi:10.1186/s12885-019-5403-0)
Supplement: Supplementary file 3 — Figure S1. Genome-wide impact of DNA methylation on gene expression. Distribution of Spearman correlation coefficients (SCC) between DNA methylation and cognate gene expression levels (59% of negative SCC; P < 2.2 × 10− 16, 1-sample proportions test). Figure S2. Higher values of principal component 2 are associated with poorer survival. Kaplan Meier (KM) curve showing patients subdivided by principal component 2 value with a cutoff of 0.095 (p = 0.01, Log-rank test). Figure S3. Heatmap showing hierarchical clustering analysis of 209 differentially-methylated CpG sites associated with 164 differentially-expressed genes. Figure S4. 2CpGs from ZNF154 and HOXD9 are epigenetically dynamic and predict prognostic. Upper panel- (A) cg01268824-ZNF154 and (B) cg22674699-HOXD9 sites are hypomethylated in normal cells, overlapping with open chromatin and active histone modification marks (H3K4me1 and H3K4me3, green peaks). ChromHMM classified (A) cg01268824 region as an active TSS (red) and (B) cg22674699 as a bivalent enhancer (dark yellow). Middle panel- KM curves evidenced that hypomethylation of CpGs located in (C) ZNF154 and (D) HOXD9 are associated with a longer overall survival. Cut-offs of 0.6188, (50th percentile of ZNF154-cg01268824) and 0.6102 (49th percentile of HOXD9-cg22674699) were used. Bottom panel- Forest plot of Cox multivariate survival analyses of methylation values of (E) ZNF154 and (F) HOXD9 CpG probes with ER status as covariate. Figure S5. Cox multivariate analyses of the 3 CpGs sites from “bottom 7” genes in BC. Forest plot of Cox multivariate survival analyses of methylation values of (A) PRCA2, (B) TDRD10 and (C) TMEM132C CpG probes with ER status as covariate. Figure S6. Prognostic signature of the 3 CpGs sites from “bottom 7” genes in BC. KM curve for the combined signature of the 3 CpGs sites correspondent to PRAC2-cg12374721, TDRD10-cg18081940 and TMEM132Ccg04475027. Low methylation levels was significantly associated with better prognosis (p = 0 [file 12885_2019_5403_MOESM3_ESM.pdf]

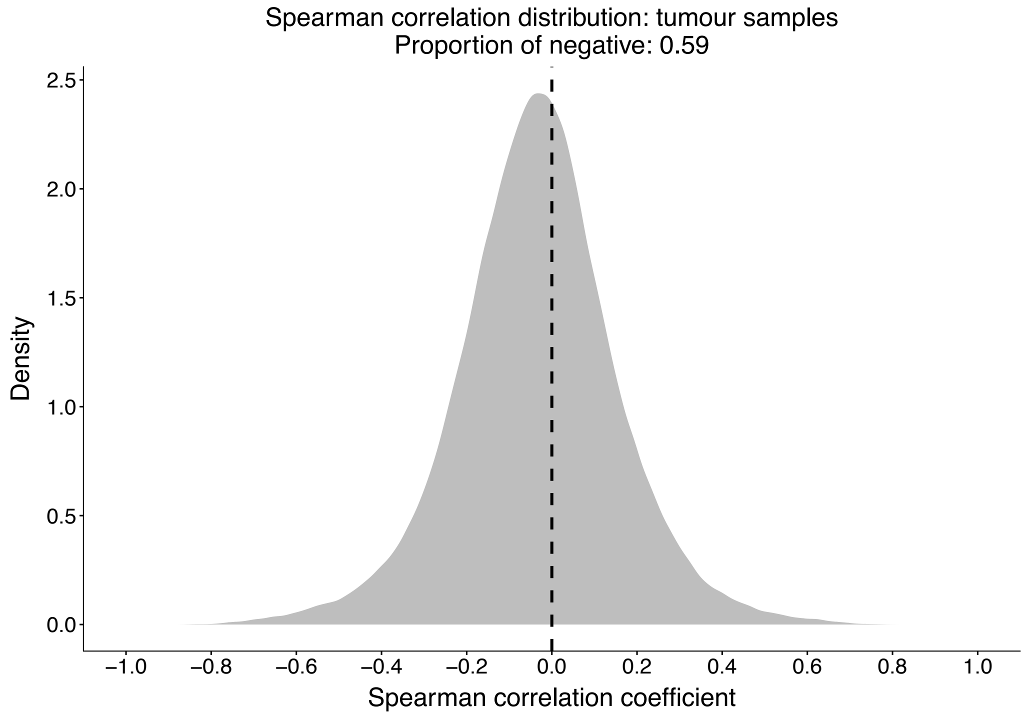

Supplementary Figure 1

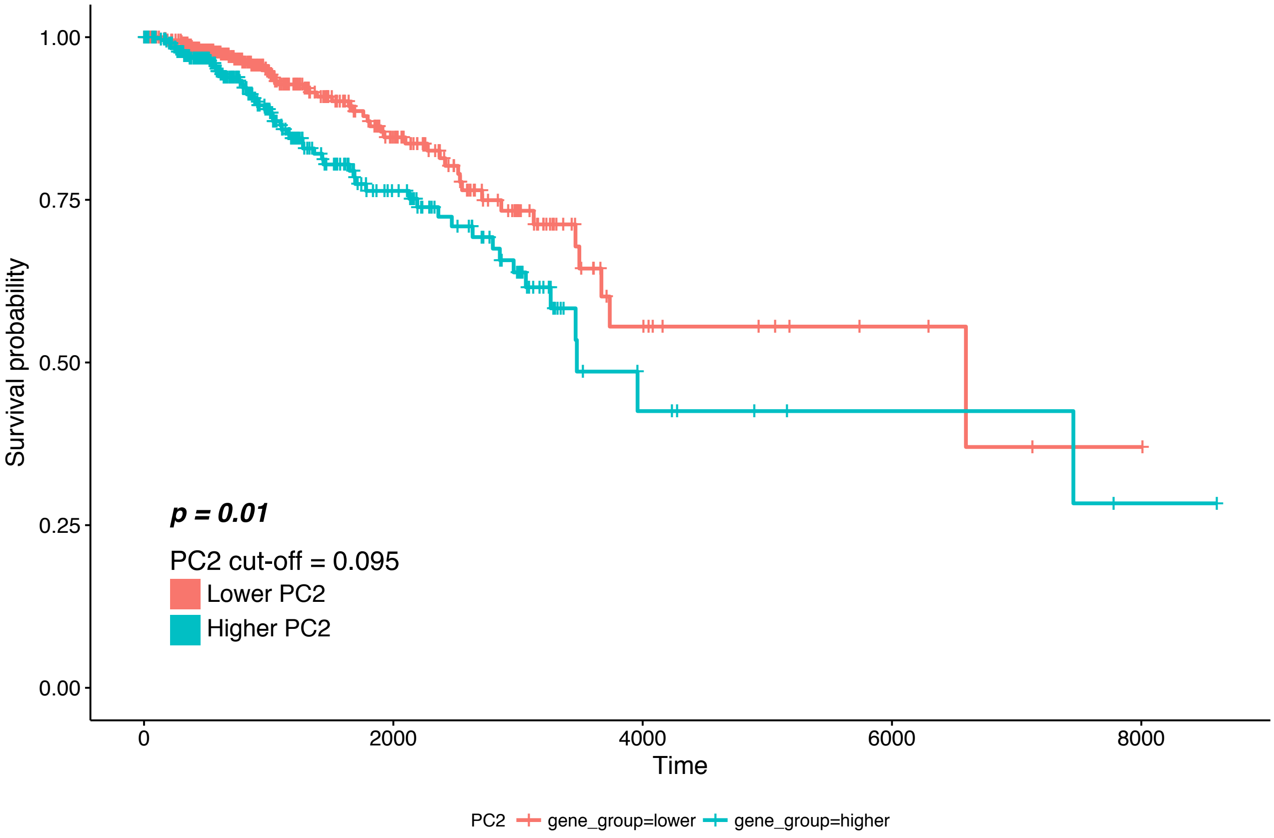

Supplementary Figure 2

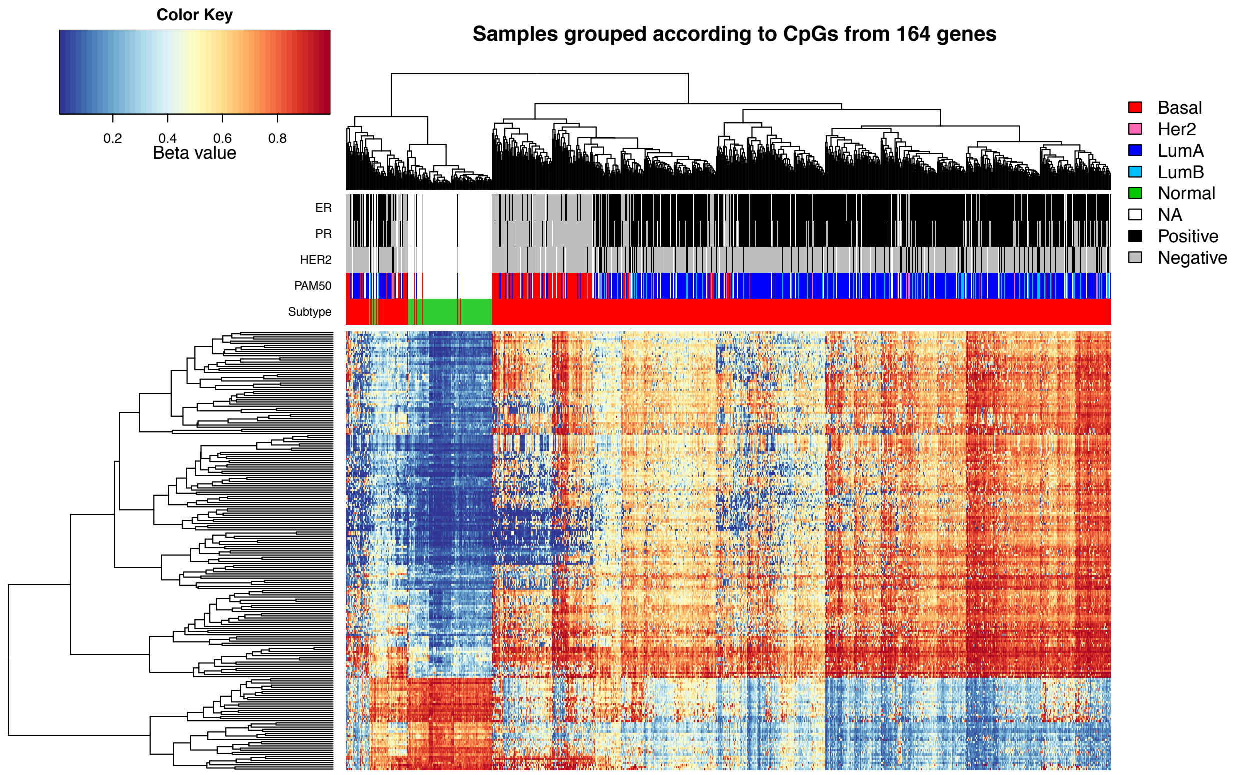

Supplementary Figure 3

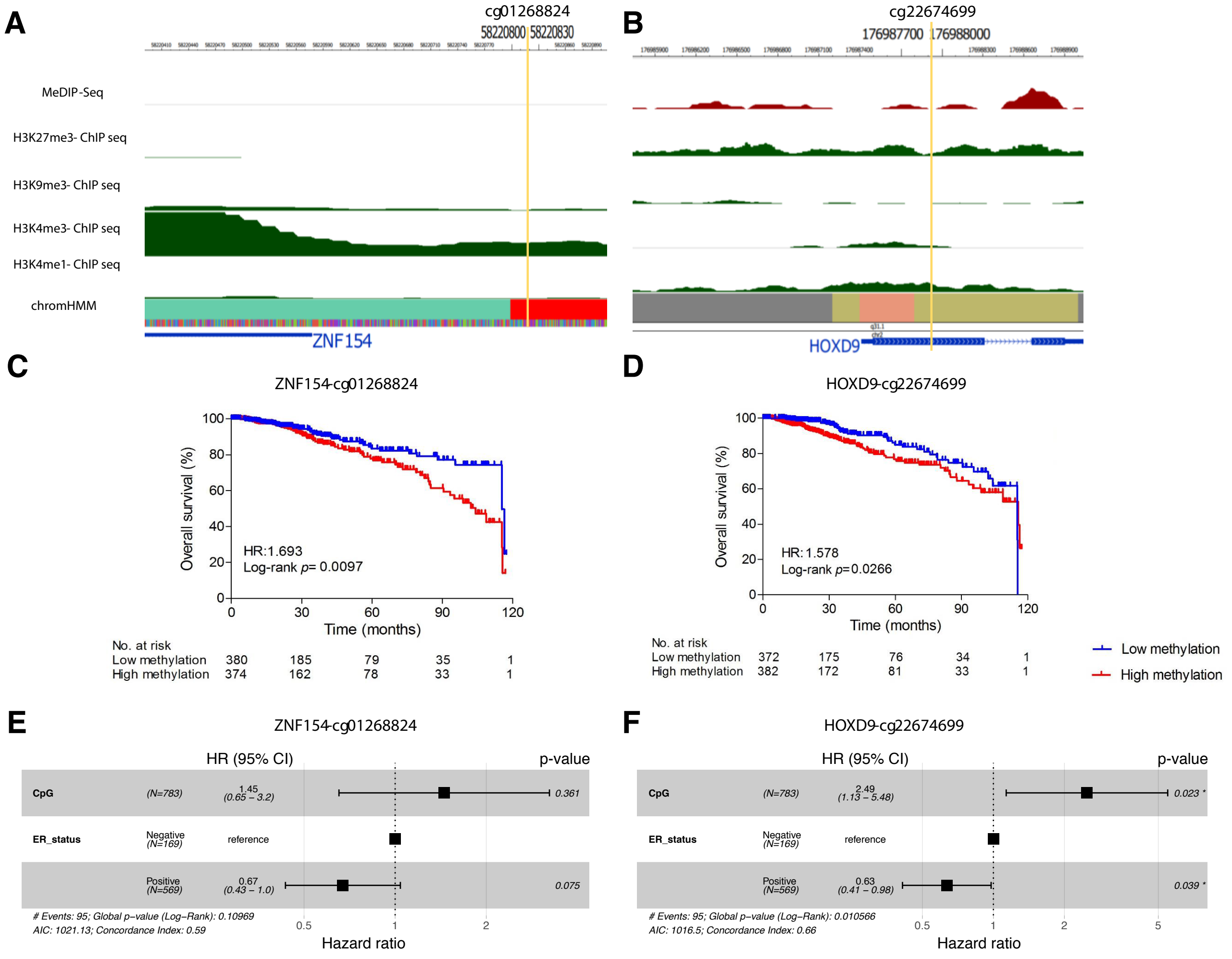

Supplementary Figure 4

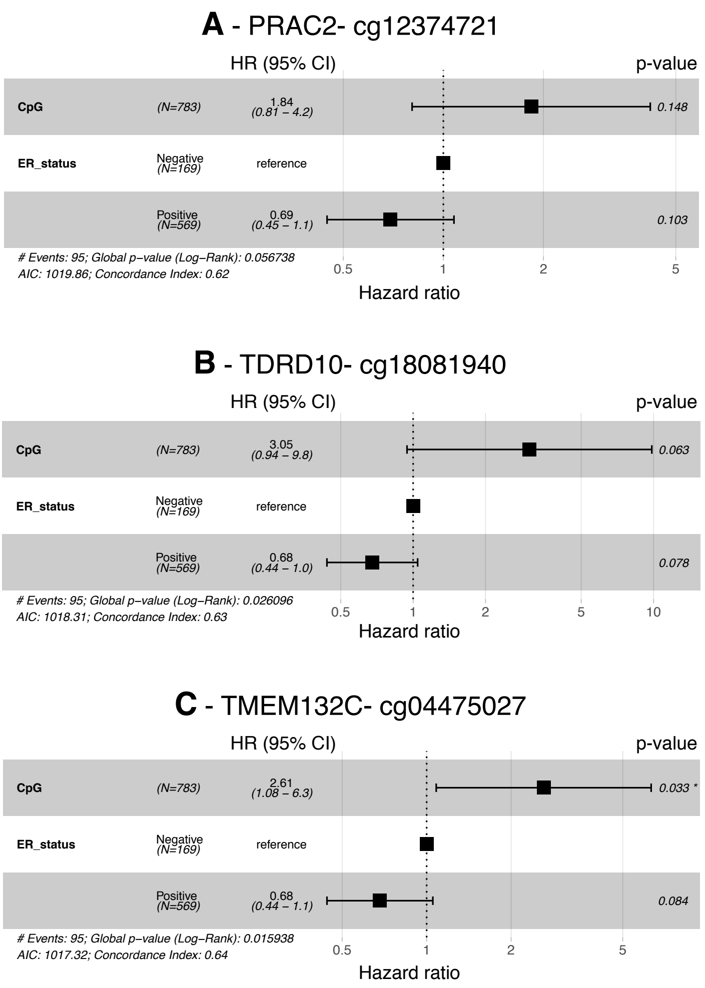

Supplementary Figure 5

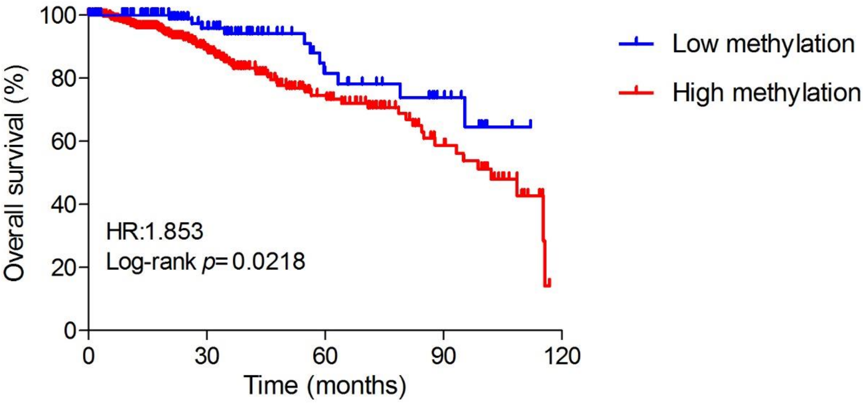

No. at risk					
Low methylation	117	64	26	12	1
High methylation	326	141	64	27	1

Supplementary Figure 6
